# ATryn^®^: 1st GE (genetically engineered) animal success story for production of a human recombinant pharmaceutical

**DOI:** 10.1186/1753-6561-8-S4-O4

**Published:** 2014-10-01

**Authors:** William Gavin

**Affiliations:** 1 rEVO Biologics, Spencer, Massachusettes, United States

## 

The development of genetically modified (transgenic) animals for the production of human recombinant therapeutic proteins has been ongoing for more than 25+ years. Only recently in the last few years have products finally been approved that can now be found commercially on the market to the benefit of the medical community and patients that receive these lifesaving therapies. rEVO Biologics has a long history in transgenic technology for this purpose and has been recognized as the leader in this field. With the approval of ATryn^® ^(antithrombin alpha) by both the EMA in 2007 and the FDA in 2009, rEVO Biologics set the standard and established significant milestones and "firsts" and is revolutionizing the application of this technology to recombinant therapeutic production. rEVO Biologics is now expanding upon this success by not only furthering the development of ATryn^® ^into subsequent larger clinical indications but also by moving additional recombinant therapeutic proteins through the pipeline from development and into early and late stage clinical trials. The following presentation will highlight the company history and evolution for rEVO Biologics over the years and the developmental path for ATryn^®^. Specifically, areas to be reviewed will include the technology, the molecular biology involved, protein expression in the mammary gland, the purification scheme, antithrombin as a molecule and its clinical development, and the regulatory path for approval of ATryn^® ^over the years. Additionally, the further clinical development of ATryn^® ^into additional clinical indications and new proteins being taken through this development, clinical trial evaluation, and regulatory approval path will be discussed.

